# Diffuse pigmented villonodular synovitis in a child – what is the best management?

**DOI:** 10.1186/1471-2474-14-S1-A6

**Published:** 2013-02-14

**Authors:** Sharmin Nizam, Lesley D Hordon

**Affiliations:** 1 Dewsbury District Hospital, Mid Yorkshire NHS Trust, WF13 4HS, UK

## Case

A 5 year old boy presented to orthopaedics in 2004 with a 3-4 month history of intermittent left knee pain. As examination was normal he required no specific treatment. In 2007, he attended A&E with a spontaneous left knee effusion with a CRP of 9 mg/l, ESR 9 mm/h, raised platelet count of 559 (10^9^/l) and mild neutrophilia of 9.23 (10^9^/l). Knee x-ray was normal. 20 mls of haemorrhagic fluid was aspirated (cultures negative). 2 months later, he was seen by rheumatologists due to fluctuant swelling, initially responding to oral non-steroidals but later requiring an intra-articular injection (20 mg triamcinolone). MRI imaging suggested PVNS.

Synovial biopsy or synovectomy was avoided due to risk of post-op stiffness. JIA was considered after 2 left knee flares following upper respiratory tract infections and transient right knee swelling. However, imaging (2007, 2009, 2012) and aspirate appearances were typical of PVNS.

Screening over 5 years revealed no evidence of uveitis. He was ANA negative and there was no history of psoriasis.

**Figure 1:**
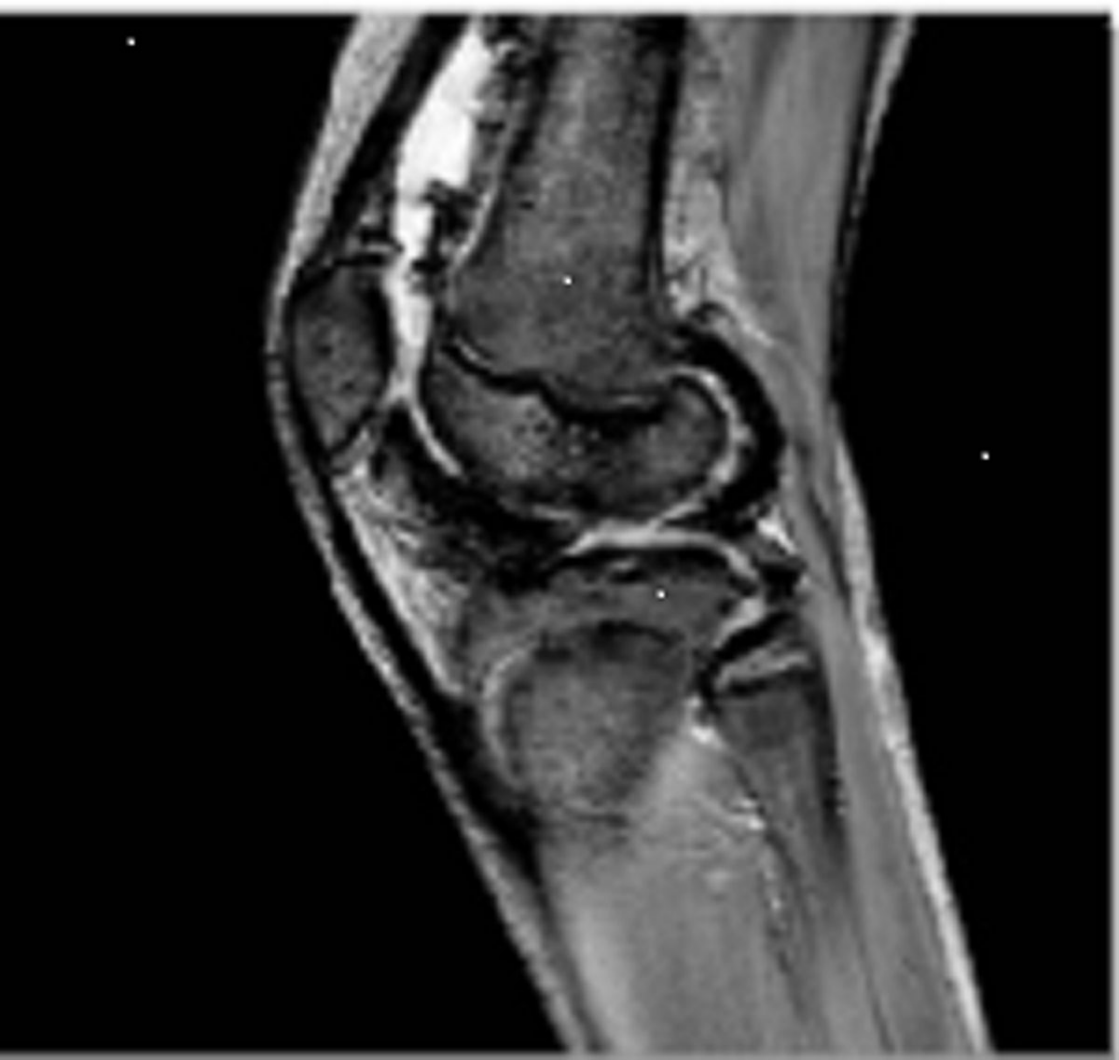
**Gradient echo MRI (2012)** Haemosiderin deposition noted

In 2009, a further intra-articular injection (20 mg triamcinolone) was required. The patient remained well and active with ibuprofen and physiotherapy until a pre-holiday flare in May 2012. His GP injected the knee, after aspirating blood, with 40 mg triamcinolone with no response. He was referred to our service for review. Repeat MRI (Figure 1) showed progressive changes. As the differential diagnosis includes recurrent haemarthrosis, synovial biopsy has been advised.

## Discussion

Pigmented villonodular synovitis (PVNS) is a rare cause of monoarthritis. Haemorrhagic effusions and typical imaging appearances (reduced signal on T1&T2-weighted MRI or hyperdensity on CT) are typical. Histology reveals hypervascular, proliferative synovium [1,2]. Insidious presentation can cause delays in diagnosis, especially in children, with potential adverse effect on prognosis [1]. Although a “benign” condition, it can cause significant joint damage and disability due to recurrent synovitis [3]. Arthroscopic/open synovectomies are recommended. Neither treatment guarantees complete eradication of affected tissue and recurrence is common [3-5]. Surgery can lead to post-op stiffness, instability and secondary osteoarthritis. Post-op adjuvant radiotherapy (low dose external beam) may reduce recurrence. Long term benefits are controversial and in paediatric cases, raise concern about risks of epiphyseal growth plate damage and post radiation sarcoma [4,5]. Evidence of benefit with anti-TNF (infliximab) and tyrosine kinase inhibitor therapies is currently limited to case reports and preliminary phase II study data. Management is thus difficult, particularly in children, because of long term complications of both the disease and treatment.
